# A Novel PTP1B Inhibitor-Phosphate of Polymannuronic Acid Ameliorates Insulin Resistance by Regulating IRS-1/Akt Signaling

**DOI:** 10.3390/ijms222312693

**Published:** 2021-11-24

**Authors:** Dan Li, Shuai Zhang, Cheng Yang, Quancai Li, Shixin Wang, Ximing Xu, Jiejie Hao, Chunxia Li

**Affiliations:** 1 Key Laboratory of Marine Drugs of Ministry of Education, Shandong Provincial Key Laboratory of Glycoscience and Glycoengineering, School of Medicine and Pharmacy, Ocean University of China, Qingdao 266003, China; ldan@stu.ouc.edu.cn (D.L.); 21170831086@stu.ouc.edu.cn (S.Z.); acheng0912@163.com (C.Y.); quancaili@126.com (Q.L.); shixin113@126.com (S.W.); xuximing@ouc.edu.cn (X.X.); 2 Laboratory of Marine Glycodrug Research and Development, Marine Biomedical Research Institute of Qingdao, Qingdao 266071, China; 3 Laboratory for Marine Drugs and Bioproducts, Pilot National Laboratory for Marine Science and Technology (Qingdao), Qingdao 266237, China

**Keywords:** low-molecular-weight polymannuronic acid phosphate, PTP1B inhibitor, anti-diabetic effects, hepatoprotective effects

## Abstract

Protein tyrosine phosphatase 1B (PTP1B) is a critical negative modulator of insulin signaling and has attracted considerable attention in treating type 2 diabetes mellitus (T2DM). Low-molecular-weight polymannuronic acid phosphate (LPMP) was found to be a selective PTP1B inhibitor with an IC_50_ of 1.02 ± 0.17 μM. Cellular glucose consumption was significantly elevated in insulin-resistant HepG2 cells after LPMP treatment. LPMP could alleviate oxidative stress and endoplasmic reticulum stress, which are associated with the development of insulin resistance. Western blot and polymerase chain reaction (PCR) analysis demonstrated that LPMP could enhance insulin sensitivity through the PTP1B/IRS/Akt transduction pathway. Furthermore, animal study confirmed that LPMP could decrease blood glucose, alleviate insulin resistance, and exert hepatoprotective effects in diabetic mice. Taken together, LPMP can effectively inhibit insulin resistance and has high potential as an anti-diabetic drug candidate to be further developed.

## 1. Introduction

Type 2 Diabetes Mellitus (T2DM) is a hyperglycemic metabolic disease that is difficult to treat and hugely impacts daily life. According to the International Diabetes Federation (IDF), approximately 451 million individuals will have diabetes, and the total number of patients is estimated to reach 693 million by 2045 [1]. Interfering with the production and function of liver insulin could lead to the development of T2DM, and insulin resistance (IR) is inextricably linked with the development of diabetes [2].

Insulin receptor substrate-1 (IRS-1) is an essential insulin receptor substrate in the insulin signaling pathway, and it plays a vital role in insulin signaling [3]. In response to insulin stimulation, the tyrosine residue site of IRS-1 is phosphorylated and is enabled to bind to phosphatidylinositol-3 kinase (PI3K) proteins containing the *Src*-homologous 2 (SH2) domain [4]. Then, PI3K recruits the protein kinase B (PKB/Akt) protein to the cell membrane, and its tyrosine residues are phosphorylated [5]. Dephosphorylation of tyrosine residues of IRS-1 and Akt may inactivate the entire process of insulin signaling, leading to insulin resistance [6]. Noteworthily, oxidative stress is the toxic effect of free radicals that occurs when the generation rate of free radicals exceeds the anti-oxidant defense system. It induces activation of pathways such as NF-κB, JNK/SAPK, and MAPK signaling [7]. These stress-activated signaling pathways also play a remarkable role in the development and progression of insulin resistance [8,9]. Moreover, endoplasmic reticulum (ER) stress is caused by the buildup of unfolded and misfolded proteins within the lumen of the endoplasmic reticulum, resulting in perturbations within the endoplasmic reticulum homeostasis [10]. Endoplasmic reticulum stress activates a sign cascade known as the unfolded protein response (UPR) and triggers a series of transcriptional and translational events that promote cell survival and adaptation [11]. Existing preclinical and clinical proof demonstrates that prolonged endoplasmic reticulum stress will increase the danger of metabolic disorders, such as T2DM, obesity, and dyslipidemia [12].

Protein tyrosine phosphatase 1B (PTP1B) is a member of the protein tyrosine phosphatase family that specifically hydrolyzes aromatic phosphates such as phosphate on tyrosine (pTyr) residues [13]. PTP1B plays a negative regulatory role on insulin signaling through dephosphorylation of tyrosine residues on insulin receptors substrates [14,15,16], since the overexpression of PTP1B in tissues and cells reduces the activity of protein tyrosine kinase (PTK) and prevents insulin from binding to insulin receptors, leading to insulin resistance and finally to T2DM [17]. In addition, mice lacking PTP1B have enhanced insulin sensitivity and increased levels of tyrosine phosphorylation of insulin resistance in the liver and muscle tissue [18]. Based on these findings, inhibition of PTP1B has emerged as an attractive therapeutic strategy for treating T2DM. The development of PTP1B inhibitors has drawn much attention for searching for more efficient PTP1B during the past few decades [19]. Unfortunately, most PTP1B inhibitors are challenging to develop into effective drug candidates due to the characteristics of poor cell permeability and low bioavailability [20,21].

Alginate is a linear heteropolysaccharide of marine origin composed of 1 → 4 linked β-d-mannuronic acid (PM) and α-l-guluronic acid (PG) [22,23]. PM and PG are a pair of epimers with completely different conformations and advanced structures [24]. Alginate and its derivatives also possess many vital biological activities, including anti-tumor [25], anti-viral [26], and immunomodulatory activities [27,28]. As a crucial part of alginate, PM-block has aroused much interest due to its essential role in metabolic diseases [29,30,31,32]. It was reported to reduce glycogen synthase kinase 3β (GSK-3β) activity by changing the phosphorylation level of GSK-3β [33], which means that MOS might have anti-hyperglycemic effects. Muhammadi et al. found that polymannuronate and its oligomer exhibit remarkably anti-obesity and anti-diabetic activities [34]. However, chemical modified derivatives of polymannuronic acid have not been reported in the treatment of T2DM. Recently, we screened the PTP1B inhibition effect of phosphorylated PG and PM with different molecular weights and phosphorus contents. The results showed that LPMP with molecular weight around 6 × 10^3^ Da and 8.7% phosphorus content had the most potent PTP1B inhibition effect (data not shown). Thus, we supposed that LPMP has the potential to exert its anti-diabetic effect by inhibiting intracellular PTP1B activity. To further investigate the anti-diabetic mechanism of LPMP, we investigated the anti-diabetic activity of the polymannuronic acid derivative in vivo and in vitro. Our results elucidated that LPMP could improve insulin resistance as a novel PTP1B inhibitor, which may contribute to the further development of LPMP as a potential therapeutic intervention for T2DM.

## 2. Results and Discussion

### 2.1. Chemical Profile of LPMP

**Figure 1:**
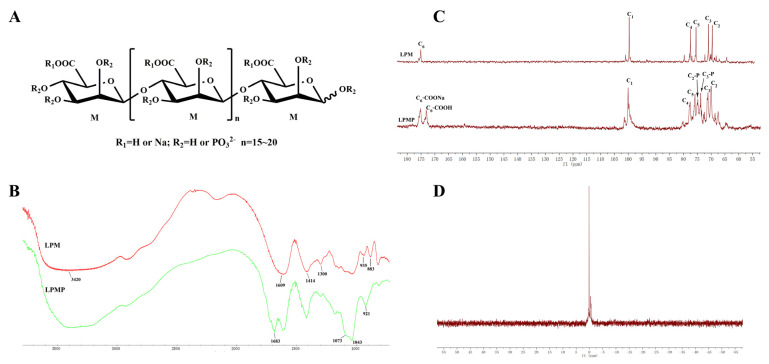
The characteristics of LPMP. (**A**) Chemical structure of LPMP. (**B**) IR spectra of LPM and LPMP. (**C**) The ^13^C-NMR spectra of LPM and LPMP. (**D**) The ^31^P-NMR spectra of LPMP.

LPMP was previously synthesized in our laboratory by the phosphoric acid urine method [35,36]. Its organophosphorus content was 8.7%, and the phosphate group substitution degree was calculated to be 0.954, with an average molecular weight of 5.8 × 10^3^ Da. The degree of polymerization (DP) of LPMP was about 15–20 (Figure 1A), and the structure of LPMP was characterized with FT-IR, ^13^C-NMR, and ^31^P-NMR. Based on FT-IR spectra (Figure 1B), the bands at 1073 and 921 cm^−1^ of LPMP are attributed to the stretching vibration of P-O-C (aliphatic) and P-O(H), respectively. Next, in the ^13^C-NMR spectrum of LPM and LPMP, the peaks at 175.15, 99.91, 77.76, 75.77, 71.30, and 69.92 ppm were attributed to C_6_, C_1_, C_4_, C_5_, C_3_, and C_2_ respectively. The peak at 74.83 ppm was attributed to C_2_-P and the peak at 73.76 ppm was attributed to C_3_-P, substituted with phosphoric acid at C_2_-OH and C_3_-OH of the LPM (Figure 1C). The relevant carbon signal values for LPM, LPMP are shown in Table 1. Furthermore, in the ^31^P-NMR spectrum, the peaks between 0–2 ppm were attributed to monophosphate groups, and no peaks attributed to diphosphates (~10 ppm) or triphosphates (~20 ppm) were found (Figure 1D). In summary, the structural characteristics of LPMP were consistent with previously published studies in our laboratory.

### 2.2. Binding of LPMP in the Active Site of PTP1B

**Figure 2:**
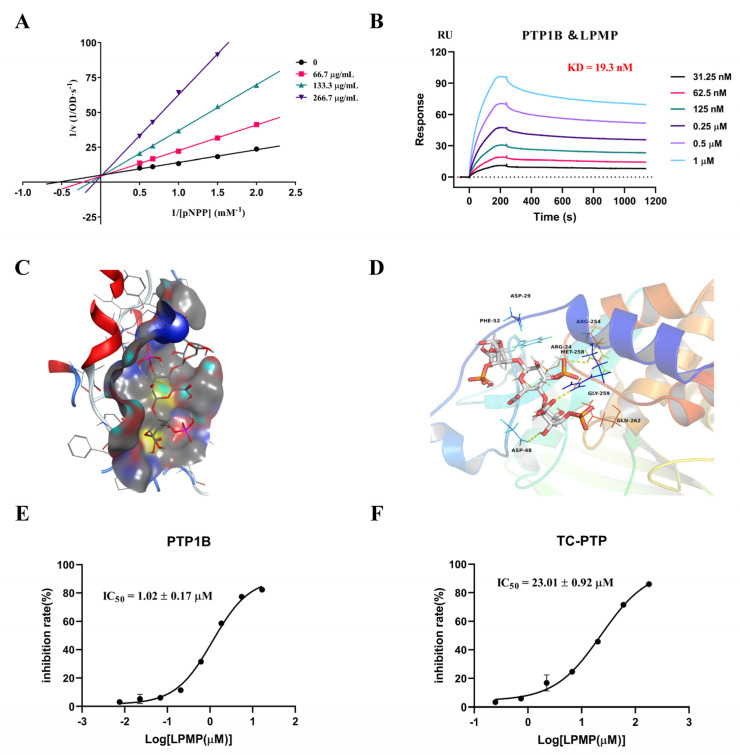
Interaction of LPMP with PTP1B. (**A**) Lineweaver Burk data analysis of LPMP inhibition of PTP1B. The reciprocal of the initial reaction rate (1/[V]) corresponds to the reciprocal of the pNPP concentration (1/[pNPP]) in the presence of different concentrations of LPMP. (**B**) Interaction of LPMP with PTP1B immobilized on the CM5 chip. (**C**) The binding site of PTP1B interacting with LPMP mode was shown as a molecular surface structure using MOE software. (**D**) A comprehensive view of the hydrogen bond and hydrophobic interactions of PTP1B-LPMP was displayed by Pymol software. (**E**) The IC_50_ value of LPMP to PTP1B. (**F**) The IC_50_ value of LPMP to TC-PTP.

To determine the inhibitory mode of PTP1B, we used the Lineweaver-Burk plot of Michaelis–Menten kinetics for analysis. The lines intersected on the y-axis and showed a dose-dependent increase in K_m_ values at constant V_max_ values, consistent with the enzyme kinetics of a competitive inhibitor, demonstrating that LPMP was a competitive inhibitor (Figure 2A). Furthermore, to evaluate whether LPMP could bind to PTP1B, we investigated the interaction of LPMP with PTP1B using a surface plasmon resonance biosensor (SPR). The assay data revealed binding of LPMP to PTP1B with KD (dissociation equilibrium constant) of 1.93 × 10^−8^ M, indicating a high affinity of LPMP for PTP1B protein (Figure 2B). In addition, molecular docking studies were performed to obtain predictions of PTP1B and LPMP interactions. As polysaccharide compounds are generally large in molecular weight, the accuracy of docking would be greatly decreased when molecular docking is performed. Considering that LPMP is a repeating polymer of PM-block phosphate, we constructed the trisaccharide structure of LPMP for molecular docking studies. The molecular docking results indicated that the activity of LPMP on PTP1B was related to the binding energy of the active site and the number of hydrogen bonds formed (Figure 2C). LPMP could form hydrogen bonds with amino acids such as Arg 254, Arg 24, Gln 262, Asp 48, and Met 258 (Figure 2D). The LPMP trisaccharide mode binding residues were essential for PTP1B enzyme activity, and the hydrogen bond between the LPMP and Asp24, Asp48, Arg254, and Gln262 of PTP1B had a particular significance [37,38,39], indicating the potent binding mode of LPMP to inhibit PTP1B. Accordingly, we determined that LPMP inhibits PTP1B by binding to the catalytic site of PTP1B. 

T-Cell Protein Tyrosine Phosphatase (TC-PTP) is a homologous protein of PTP1B with a high degree of homology in the catalytic structural domain (74% of the amino acid sequence) [40]. Meanwhile, TC-PTP is a phosphatase associated with the regulation of T-cell activation and plays an essential role in human hematopoiesis [41]. Simultaneous pharmacological inhibition of PTP1B and TC-PTP is believed to cause a range of side effects [13]. In the present study, we found that the IC_50_ of LPMP for PTP1B is 1.02 ± 0.17 μmol·L^−1^ (Figure 2E) and that the IC_50_ for TC-PTP was approximately 23-fold higher than that of PTP1B (Figure 2F). The inhibition of LPMP for TC-PTP was much lower than that of PTP1B, and we speculate that LPMP might have a certain selectivity for PTP1B. 

### 2.3. Effect of Cell Permeability and Intracellular Enzyme Activity

**Figure 3:**
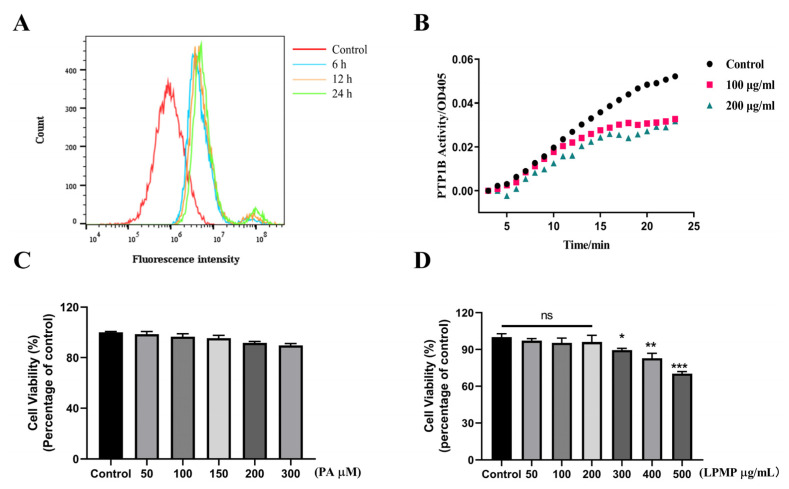
The cellular permeability and toxicity of LPMP in HepG2 hepatocytes. (**A**) HepG2 cells were assayed by flow cytometry for fluorescence levels of FITC after various periods of treatment with LPMP-FITC (200 μg·mL^−1^). (**B**) The effect of LPMP on the catalytic activity of PTP1B protein in HepG2 cells. The intracellular PTP1B protease activity was measured at different administration periods of 100 and 200 μg·mL^−1^ LPMP. (**C**) Cytotoxicity of sodium palmitate on HepG2 cells in the absence or presence of 50, 100, 150, 200, and 300 μg·mL^−1^ of PA for 24 h at 37 °C. (**D**) Cytotoxicity of LPMP on HepG2 cells in the absence or presence of 50, 100, 200, 300, 400, and 500 μg·mL^−1^ of LPMP for 24 h at 37 °C. Data are presented as means ± SEM (*n* = 5). * *p* < 0.05, ** *p* < 0.01, *** *p* < 0.001 versus control group.

Although PTP1B inhibitors have been developed for many years, no PTP1B inhibitors have been marketed. Many compounds are promising in vitro inhibitory effects on PTP1B, but the low cellular permeability limits their future applications [42]. Cellular permeability is a vital prerequisite for LPMP to exert its PTP1B inhibitory effect in cells. LPMP was FITC-labeled by chemical coupling, and the measured labeling rate was 2.3‰ (see Supplementary Information). The fluorescence intensity of LPMP-FITC was measured by flow cytometry to determine the cell permeability of LPMP. The results showed that the fluorescence intensity of LPMP increased at 6 h compared with the control group, indicating that LPMP could be taken up by HepG2 cells (Figure 3A). Meanwhile, further investigation was needed to investigate whether LPMP could affect the intracellular PTP1B viability after being taken up by cells. Therefore, intracellular PTP1B protease activity was measured at different periods of LPMP treatment (100 and 200 μg·mL^−1^). We found that the intracellular PTP1B enzyme activity declined with the increasing duration of LPMP treatment (Figure 3B), suggesting that LPMP could affect intracellular PTP1B activity. 

### 2.4. Effect of Sodium Palmitate (PA) and LPMP on the Viability of HepG2 Cells

The CCK-8 cell viability assay kit was used to detect the effect of LPMP and PA on HepG2 cell viability. Since subsequent experiments required palmitate to construct a model of HepG2 insulin-resistant cells, we tested the effect of PA on the viability of HepG2 cells and no remarkable effect of PA on the cell viability of HepG2 cells (Figure 3C). Based on the results of the experiments in the literature [43], we constructed a model of insulin resistance by using 200 μM PA in the subsequent experiments. For LPMP, HepG2 cells were co-cultured with LPMP at the designated concentrations for 24 h. We found that the cell viability was close to that of the control group, when the LPMP concentration was less than 200 μg·mL^−1^. As the concentration of LPMP increased, there was a slight decline in cell viability. When the LPMP concentration reached 400 μg·mL^−1^, the cell viability remained around 80%, indicating that LPMP was relatively non-toxic to HepG2 cells (Figure 3D). These results provided an indicative concentration for further experiments, and subsequent experiments were conducted using concentrations less than 200 μg·mL^−1^.

### 2.5. LPMP Increases Insulin Sensitivity in HepG2 Cells

**Figure 4:**
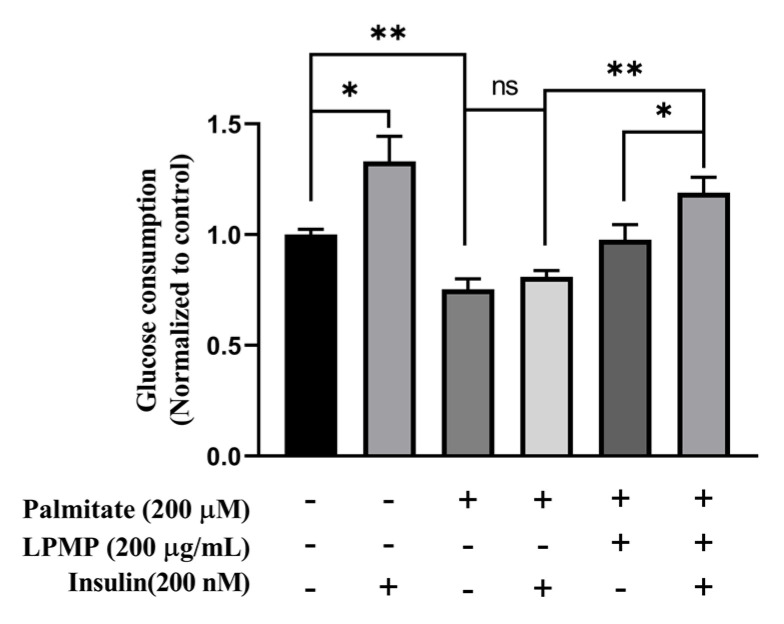
Effect of LPMP on improving insulin resistance in HepG2 cells. In the absence or presence of LPMP, HepG2 cells were treated with 200 μM PA for 24 h. Then, cells were incubated for a further 4 h with or without 200 nM insulin at 37 °C. Cellular glucose consumption was determined by detecting the reduction of glucose in the culture medium. Data are presented as means ± SEM (*n* = 5). * *p* < 0.05, ** *p* < 0.01.

Given the excellent PTP1B binding and inhibitory activity of LPMP, we investigated the anti-diabetic mechanism of LPMP in vitro using a model of HepG2 insulin-resistant cells generated by palmitate. Since the literature previously reported that 200 μM PA could induce insulin resistance in hepatocytes [43,44,45], we also found that PA had little effect on hepatocyte viability (Figure 3C). Therefore, HepG2 cells were co-incubated with LPMP containing 200 μM PA for 24 h. Next, the medium containing 200 nM insulin was added, and incubation continued for 4 h. The amount of cellular glucose consumption was subsequently determined by detecting the reduction in glucose. According to the results, insulin stimulation could obviously increase cellular glucose consumption compared to the control group (Figure 4). More importantly, cellular glucose consumption was not much changed after insulin stimulation in the model group, indicating that the cells were in an insulin-resistant (IR) state. In addition, PA treatment decreased cellular glucose consumption by 1/3rd as compared to the control group. The above results denoted that 200 μM of PA could successfully induce an insulin resistance model. We then explored the effects of LPMP on cellular glucose consumption and insulin sensitivity. After treatment of LPMP, the insulin sensitivity was enhanced, and glucose consumption was approximately 30% higher than that of PA-treated cells. Furthermore, we found that insulin could increase the glucose consumption of LPMP treatment to some extent compared to the model group. In conclusion, our experiments showed that LPMP markedly ameliorated insulin sensitivity in HepG2 cells.

### 2.6. LPMP Alleviates Insulin Resistance by Activating Insulin Signaling Pathways

**Figure 5:**
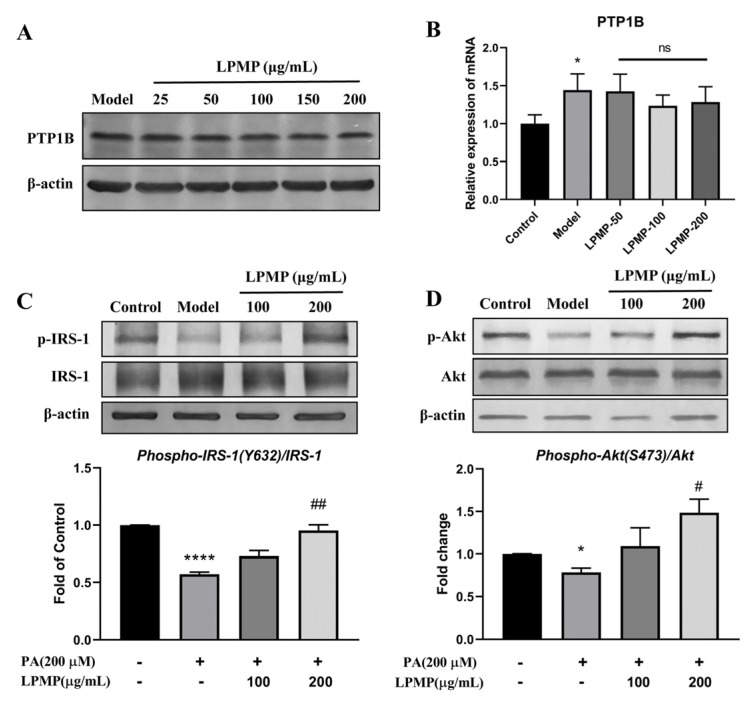
Effect of LPMP on the insulin signaling pathway. (**A**) Effect of LPMP on cellular PTP1B protein expression. (**B**) PTP1B mRNA level was measured by RT-PCR. After treatment with different concentrations of LPMP, cells were then continued to be treated with 100 nM insulin in serum-free MEM for 10 min. Phosphorylation levels of IRS-1 (**C**) and Akt (**D**) were detected by immunoblotting. The band density was quantified and *β-actin* was used as a standard. The results shown are means ± SEM (*n* = 3). * *p* < 0.05, **** *p* < 0.0001 versus control group; # *p* < 0.05, ## *p* < 0.01 versus model group.

We have confirmed that LPMP could improve cellular insulin sensitivity to some extent. However, the underlying mechanism of LPMP affecting insulin sensitivity is unclear. Although we found that LPMP could affect the enzymatic activity of intracellular PTP1B, it is unclear whether this effect was achieved by affecting PTP1B expression or by directly inhibiting PTP1B activity. Therefore, the effect of LPMP on cellular PTP1B protein and mRNA levels requires further validation. After treatment of different concentrations of LPMP for 24 h, we found no notable changes in PTP1B protein and mRNA levels in the cells (Figure 5A,B). We confirmed that inhibition by LPMP occurs through direct bind on the catalytic site of PTP1B.

Meanwhile, insulin signaling is initiated when insulin binds to the extracellular α-subunit of the insulin receptor, and downstream phosphorylation of IRS-1 at key tyrosine residues is critical for signal transduction [46,47]. PTP1B plays a negative regulatory role in insulin signaling through dephosphorylation of IRS-1 tyrosine. Therefore, we explored the effect of LPMP on the insulin signaling pathway. After inducing insulin resistance in HepG2 cells by PA, cells were stimulated with 100 nM of insulin for 10 min after 24 h of LPMP treatment. The expression of phosphorylated IRS-1 (Y632) and AKT (S437) was significantly upregulated compared to the PA-treated group (Figure 5C,D). Our results confirmed that LPMP could activate insulin sensitivity by upregulating insulin signaling through activation of the IRS-1/AKT signaling pathway.

### 2.7. LPMP Improves Cellular Oxidative Stress Levels

**Figure 6:**
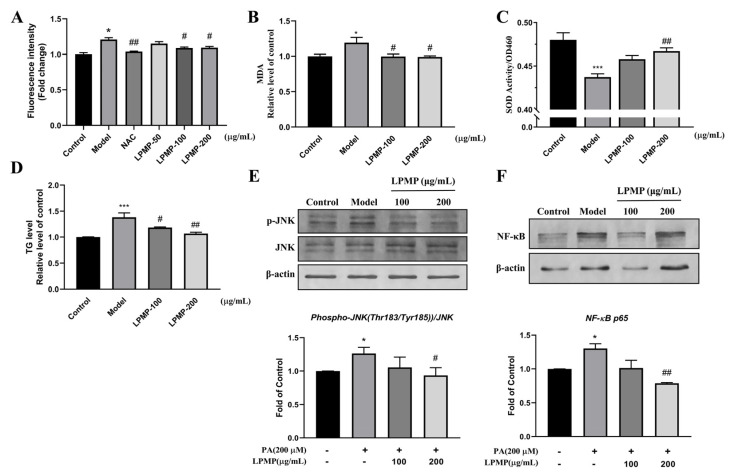
Effect of LPMP on intracellular oxidative stress in HepG2 cells. (**A**) Detection of ROS levels by in situ DCFH-DA probe. Model group (200 μM PA), positive drug group (100 μg·mL^−1^ NAC). Determination of (**B**) MDA and (**C**) SOD content. (**D**) Measurement of intracellular TG content. (**E**) Phosphorylation levels of JNK protein between the different treatment groups. (**F**) Variation in NF-κB protein levels between treatment groups. The band density was quantified and *β-actin* was used as a standard. The results shown are means ± SEM (*n* = 3). * *p* < 0.05, *** *p* < 0.001 versus control group; # *p* < 0.05, ## *p* < 0.01 versus model group.

It has been reported that increased reactive oxygen species (ROS) and oxidative damage occurred before the development of insulin resistance, indicating that reactive oxygen species play a causative role in developing insulin resistance [48]. ROS accumulation inhibits phosphatase activity and further blocks insulin signaling. As shown in Figure 6A, ROS production in HepG2 cells was dramatically increased after PA treatment (200 μM). The level of intracellular reactive oxygen species reduced after LPMP treatment (100 and 200 μg·mL^−1^), with 100 μg·mL^−1^ N-Acetylcysteine (NAC) as a positive control. Superoxide dismutase (SOD) activity was measured to determine the ability of the drug to scavenge oxygen free radicals [49]. On the other hand, the Malondialdehyde (MDA) level was measured to indirectly determine the extent of a free radical attack on cells [50]. In the model group, we found that SOD activity was dramatically decreased, and MDA content was observably increased, indicating that the cells were in a state of oxidative stress (Figure 6B). After treatment with different concentrations of LPMP, the MDA content decreased drastically while SOD activity increased, indicating that LPMP could relieve oxidative stress caused by PA (Figure 6C). At the same time, we found that LPMP could reduce the intracellular triglyceride (TG) content, illustrating that LPMP has hypolipidemic activity (Figure 6D).

Meanwhile, the C-Jun N-terminal kinase (JNK) and nuclear factor-κB (NF-κB) have also been reported to inhibit phosphatase activity, and they are involved in the induction of insulin resistance [8,51,52,53]. It has been reported that inhibition of the JNK pathway in the liver improves insulin resistance and reduces blood glucose levels in a genetic model of T2DM (db/db) mice [54,55]. As shown in Figure 6E, palmitate induced phosphorylation of JNK by 1.25-fold. Treatment of the cells with LPMP (100 and 200 μg·mL^−1^) could downregulate the phosphorylation of JNK (decreased to 1.2-fold). Free fatty acids have been reported to activate NF-κB and exacerbate insulin resistance in cells [56,57]. As shown in Figure 6F, palmitate could lead to an elevated expression of the NF-κB, and treatment with LPMP (100 and 200 μg·mL^−1^) resulted in a 40% decrease in NF-κB levels. Our results revealed that LPMP could alleviate insulin resistance by reducing intracellular ROS levels, JNK phosphorylation levels, and NF-κB expression. These results revealed that LPMP could enhance insulin sensitivity via the ROS-JNK/NF-κB pathway and lead to insulin signaling recovery.

### 2.8. LPMP Alleviates Endoplasmic Reticulum (ER) Stress Levels

**Figure 7:**
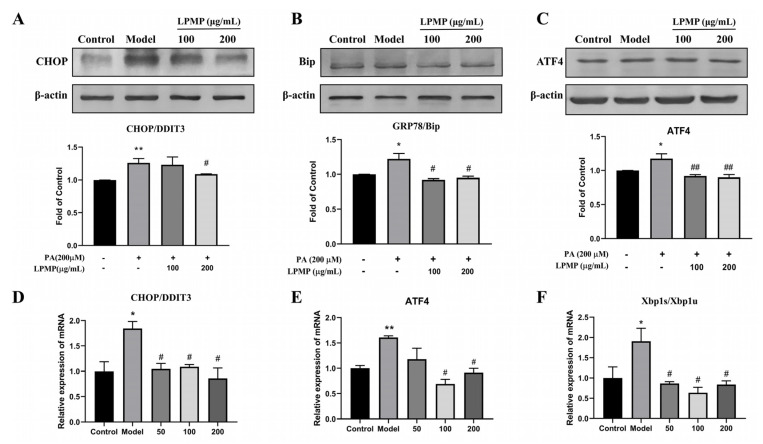
Effect of LPMP on endoplasmic reticulum stress. Treatment of HepG2 cells with different concentrations of LPMP for 24 h. (**A**) *CHOP*, (**B**) Bip, and (**C**) *ATF4* protein was determined by immunoblotting. The band density was quantified and *β-actin* was used as a standard. The mRNA expression level of (**D**) *CHOP*, (**E**) *ATF4*, and (**F**) *Xbp1s/Xbp1u* was quantified by RT-PCR. The results shown are means ± SEM (*n* = 3). * *p* < 0.05, ** *p* < 0.01 versus control group; # *p* < 0.05, ## *p* < 0.01 versus model group.

Palmitate was reported to induce endoplasmic reticulum stress (ER stress) via the *ATF4/CHOP* pathway in HepG2 hepatocytes [58]. Endoplasmic reticulum stress could induce the development and progression of metabolic diseases, such as T2DM [59]. At the same time, pathological conditions of IR further exacerbate endoplasmic reticulum stress, forming a vicious cycle [11]. Palmitate treatment increased protein expression of ER stress biomarkers, including GRP78, *CHOP*, and *ATF4*, which are representative molecules of the unfolded protein response (UPR) pathway. We found that LPMP treatment remarkably downregulated the expression of ER stress biomarkers induced by palmitate (Figure 7A–C). Consistent with protein expression, PA could upregulate the mRNA level of *CHOP*, *ATF4*, and *Xbp1s/Xbp1u*. After further treatment with LPMP, the mRNA levels of all these unfolded protein biomarker molecules decreased (Figure 7D–F). In conclusion, our results indicated that LPMP could reduce the endoplasmic reticulum stress response by regulating the UPR pathway.

### 2.9. Effect of LPMP on Blood Glucose and Insulin Sensitivity in T2DM Mice

**Figure 8:**
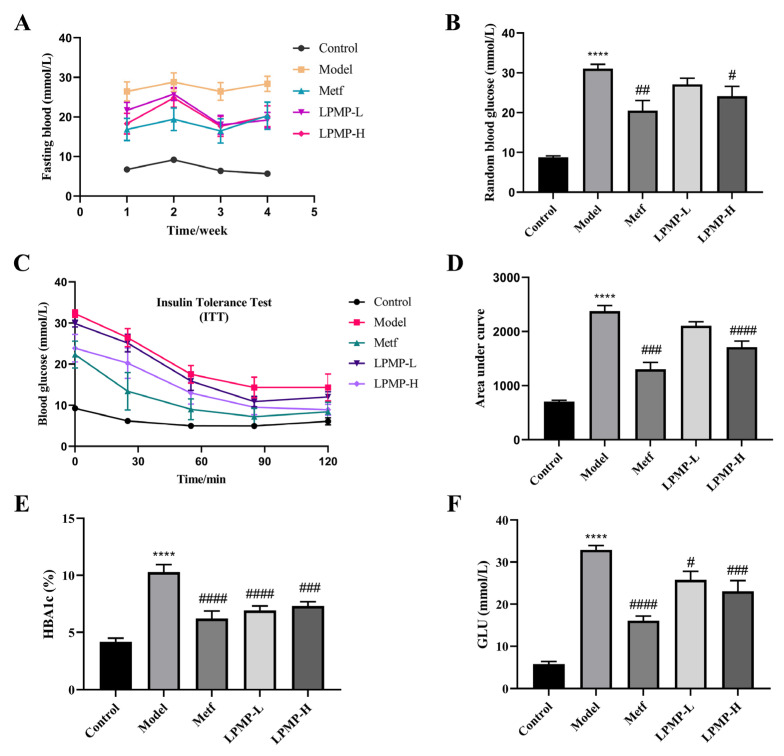
Effects of LPMP treatments on (**A**) fasting blood glucose levels, (**B**) random blood glucose levels, (**C**) insulin tolerance test, and (**D**) areas under the curve of insulin tolerance test of each group in diabetic mice. (**E**) HbA1c and (**F**) glucose concentrations. These results shown are means ± SEM (*n* = 8). **** *p* < 0.0001 versus control group; # *p* < 0.05, ## *p* < 0.01, ### *p* < 0.001, #### *p* < 0.0001 versus model group.

Elevated blood glucose and increased systemic insulin resistance are closely associated with the development of T2DM. Therefore, we constructed a mouse model of T2DM and evaluated the effects of LPMP on blood glucose levels and insulin sensitivity. The results of fasting glucose and random blood glucose measurements showed that the T2DM model was successfully constructed. As shown in Figure 8A,B, LPMP could significantly reduce fasting hyperglycemia levels at weeks 3 and 4 compared to the model group, suggesting that LPMP could improve blood glucose levels in T2DM mice. At the end of the trial, the insulin tolerance test (ITT) results demonstrated that LPMP treatment effectively improved insulin sensitivity in T2DM mice (Figure 8C,D). Administration of LPMP and metformin effectively reduced glycosylated hemoglobin levels (Figure 8E) and serum blood glucose levels (Figure 8F) compared to the model group, manifesting that LPMP has a long-term effect alleviating hyperglycemia. These analyses confirmed that LPMP has an anti-diabetic effect by regulating blood glucose and modulating insulin sensitivity.

### 2.10. Protective Effect of LPMP on the Liver of T2DM Mice

**Figure 9:**
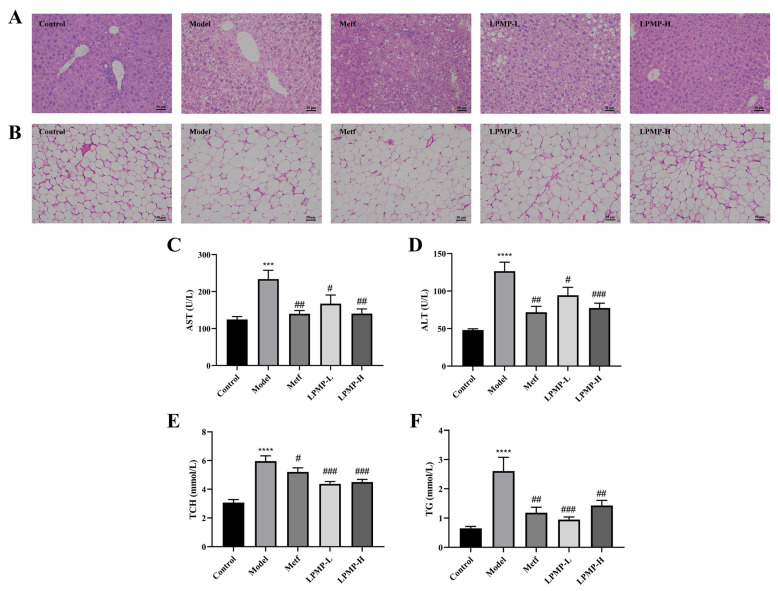
Effect of LPMP on the pathological changes of liver (**A**) and adipose (**B**) tissue. H&E staining was conducted to observe the pathological changes under 200× magnification. Control: 5 mL/kg/day water; Model: 5 mL/kg/day water; Metf: 225 mg/kg/day metformin; LPMP-L: 20 mg/kg/day LPMP; LPMP-H: 100 mg/kg/day of LPMP. Serum levels of AST (**C**), ALT (**D**), TCH (**E**), and TG (**F**) in diabetic mice. The results shown are means ± SEM (*n* = 8). **** *p* < 0.001, *** *p* < 0.001 versus control group; # *p* < 0.05, ## *p* < 0.01, ### *p* < 0.001 versus model group.

The liver was a central insulin organ, and it plays a crucial role in the homeostasis of blood glucose in the body. Adipocytes secrete pro-inflammatory factors that induce chronic inflammation and interfere with insulin signaling, eventually leading to the development of T2DM. Compared to the control group, many fat vacuoles and irregular hepatic cord structures could be observed in liver tissue sections of the model group (Figure 9A). However, the morphological structure of hepatocytes in T2DM mice was remarkably restored after treatment with different concentrations of LPMP. Additionally, the permeability of the liver cell membrane is changed when the liver is damaged, and aspartate aminotransferase (ALT) and alanine aminotransferase (AST) leak out of the cells into the circulating blood. Therefore, ALT and AST are important biomarkers to determine the degree of liver damage [60]. Compared to the control group, both AST (Figure 9C) and ALT (Figure 9D) levels were vastly higher in the serum of the model mice. After treatment with metformin and LPMP, there was a much decrease in serum AST and ALT levels. These results indicated that LPMP has a notable hepatoprotective effect. 

Meanwhile, the analysis of adipose histopathological sections showed that the proportion of adipocytes in the model group was markedly higher than the control group (Figure 9B). However, after treatment with metformin and LPMP, there was a marked reduction in the adipocyte’s ratio, suggesting that LPMP has a certain hypolipidemic effect. In addition, hyperlipidemia was a severe complication of IR, characterized by increased serum lipid accumulation. The levels of serum total cholesterol (TCH) and triglyceride (TG) of mice in the model group were considerably higher than those in the normal-fed mice (Figure 9E,F). In summary, these results indicated that LPMP has a certain hepatoprotective effect, reducing lipid accumulation in the liver, in adipose tissue.

**Figure 10:**
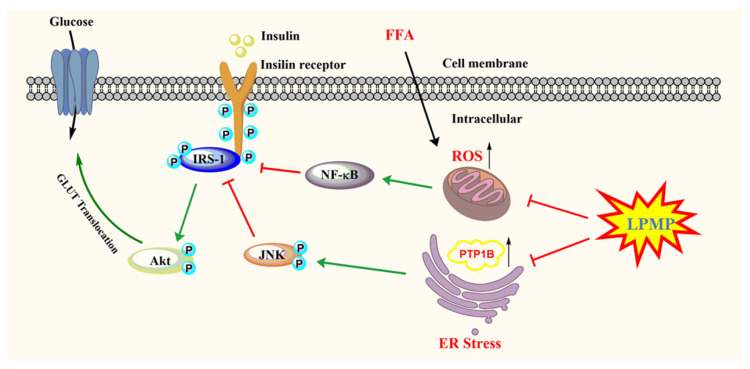
The proposed molecular mechanisms of the anti-diabetes effect of LPMP.

In summary, we identified LPMP as a PTP1B inhibitor and investigated its underlying anti-diabetic mechanism. Currently approved drugs on the market for the treatment of T2DM include metformin, sulfonylureas, GLP-1 agonists, DPP-4 inhibitors, and SGLT1/2 inhibitors, all of which are associated with more or fewer side effects [61]. Additionally, PTP1B is a promising drug target for the treatment of T2DM and obesity. LPMP is a novel PTP1B inhibitor with good cellular permeability compared to conventional PTP1B inhibitors and has initially exhibited high selectivity. Both of these characteristics would make it possible for LPMP to avoid the obstacles faced by conventional PTP1B inhibitors and have the potential to be further developed as an anti-diabetic drug. Our study found that LPMP could restore the phosphorylation level of IRS-1 by inhibiting PTP1B, thus activating the insulin signaling pathway and increasing the phosphorylation level of Akt. The results of animal trials suggested that LPMP has hepatoprotective effects. Moreover, hepatic oxidative stress and endoplasmic reticulum stress are not only closely related to T2DM and but can lead to liver damage and induce hepatocyte apoptosis. Further investigation indicated that LPMP could ameliorate sodium palmitate-induced oxidative stress and endoplasmic reticulum stress, thus partially explaining the mechanism of the hepatoprotective effect of LPMP (Figure 10). Therefore, LPMP has huge potential for further development as an anti-diabetic drug.

This study has some limitations. Firstly, we initially found that LPMP is selective, but other PTPases such as SHP-1, SHP-2, LAR, LRP are also present in the cells, and further studies are needed to investigate the inhibitory effect of LPMP on them. Secondly, the effect of LPMP on plasma insulin levels and glucagon levels in T2DM mice needs to be further clarified. The anti-diabetic activity of LPMP in db/db mice also needs to be addressed in future study.

## 3. Conclusions

In the present study, we first identified LPMP as a specific novel PTP1B inhibitor with good cell permeability and anti-diabetic effect. More importantly, LPMP could activate insulin signaling and enhance insulin sensitivity via the PTP1B/IRS-1/Akt pathway in HepG2 insulin-resistant cells. Meanwhile, LPMP could relieve oxidative stress and endoplasmic reticulum stress caused by sodium palmitate and protect hepatocytes. In vivo experiments confirmed that LPMP administration effectively reduced hyperglycemia and hyperlipidemia, improved insulin resistance, exerted hepatoprotective effects, and recovered damaged liver to the healthy level in T2DM mice. Therefore, LPMP has huge potential for further development as an anti-diabetic drug with high selectivity for PTP1B.

## 4. Materials and Methods

### 4.1. Preparation of LPMP Labeled with FITC

LPMP was synthesized in our laboratory by the phosphoric acid urine method [35,62]. Moreover, the FITC-labeled LPMP was prepared with slight modifications according to the published literature [63]. Briefly, the LPMP (100 mg) was dissolved in 5 mL of 10% acetic acid containing 0.5 mol·L^−1^ hexane diamine. After thorough stirring, 500 mg of sodium cyanoborohydride was added to the solution and stirred in the dark for 36 h. The unreacted hexamethylenediamine acetate and NaBH_3_CN were then removed with a 1000 Da dialysis bag from the reaction solution. The pH was then adjusted to 8.5 with 0.5 mol·L^−1^ NaHCO_3_. Then, FITC (Sigma-Aldrich, St. Louis, MO, USA) methanol solution (10 mg/mL) was added, and the reaction was performed for 24 h at 37 °C in the dark. Subsequently, the product was precipitated with ethanol, and the precipitate was collected by centrifugation. Finally, the LPMP-FITC was collected by freeze-drying. The method for measuring the FITC labeling rate was available in the Supplementary Information.

### 4.2. Analytical Methods of LPMP

The structure of LPMP was characterized by nuclear magnetic resonance (NMR) and Fourier transform infrared spectroscopy (FT-IR) analysis. LPMP was ground with KBr in a quartz mortar and subsequently compressed into the tablet. It was then examined using a Thermo Nicolet Nexus 470 infrared spectrometer with a scanning range of 400–4000 cm^−1^. The NMR spectra of the LPMP (30 mg) dissolved in D_2_O were examined using an Agilent DD2 superconducting NMR spectrometer. The content of organophosphorus in LPMP was determined using the phosphomolybdate method as previously reported [64].

### 4.3. Enzyme Activity Inhibition and Kinetics Assay In Vitro

Protein tyrosine phosphatase inhibition assay using pNPP as reaction substrate. The assay buffer contains 25 mM Hepes, 1 mM EDTA and DTT, and 50 mM NaCl. The assay procedure was as follows: 3 μL of the designated concentration LPMP in buffer solution was added to 97 μL of 2.5 μg·mL^−1^ PTP1B or TC-PTP (SinoBiological, Beijing, China) in buffer solution. Then, 50 μL of 6 mM pNPP was mixed into the reaction solution and incubated for 20 min at 37 °C. The final reaction was discontinued by adding 50 μL of 3 M NaOH to the mixture. The OD at 405 nm was recorded using Spark 10M (Tecan Trading AG, Männedorf, Switzerland), and the IC_50_ was calculated based on non-linear fit.

For kinetics assay, Lineweaver–Burk plot data analysis of Michaelis–Menten kinetics was used to determine the type of LPMP inhibition of PTP1B. Values of 1/[S] (mM^−1^) and 1/[V] (1/OD·s^−1^) were converted by substrate concentration and initial reaction rate, respectively, followed by the use of ddH_2_O as control. The type of enzyme inhibition was characterized by the intersection of the linear-regression curves and the change in K_m_ values.

### 4.4. Surface Plasmon Resonance Assay

To detect the interaction between LPMP and PTP1B, we conducted Surface plasmon resonance (SPR) experiments using Biacore T200. PTP1B and TC-PTP protein (10570-HNCB, SinoBiological, Beijing, China) were immobilized on a CM5 microchip via a protein coupling kit (BR-1000-50, GE Healthcare Life Sciences, Mississauga, Ontario, Canada). Briefly, the mixture solution of EDC [*N*-ethyl-*N*′-(dimethylaminopropyl)-carbodiimide] and NHS [N-hydroxysuccinimide] was injected and flowed through the chip to complete the activation of the CM5 chip. PTP1B and TC-PTP protein were diluted to 15 μg/mL with sodium acetate buffer (pH 5.5), followed by running a ligand-protein coupling program that ultimately coupled approximately 1200 response units (RUs) on the chip. The CM5 chip was then flowed through with ethanolamine (pH 8.5) to elute excess active esters from the chip, thereby reducing non-specific binding. The chip reference channel also completed the activation, immobilization, and elution steps.

For kinetic analysis of tested compounds and their corresponding protein interactions, the LPMP was dissolved in distilled water and configured to 2 mM, then diluted the LPMP to the designated concentrations with Hepes buffer. Different concentrations of LPMP were injected into the chip immobilization channel and the reference channel, with an allotted dissociation time of 900 s. The kinetic evaluation was carried out using Biacore T200 evaluation software subtracted reference data and the solvent correction. A nonlinear curve fitting (global fitting, 1:1 model) was then applied to the entire dataset.

### 4.5. Molecular Docking of LPMP at the PTP1B Binding Site

Docking studies were carried out with Molecular Operating Environment (MOE, Chemical Computing Group Inc., Montreal, QC) software. The PTP1B (Homo sapiens, NM_002827.4) protein sequence was obtained by searching the National Center for Biotechnology Information (NCBI, https://www.ncbi.nlm.nih.gov/, accessed on 28 June 2021). We then searched the Protein Data Bank (PBD, http://www1.rcsb.org/, accessed on 28 June 2021) and found a structure that was highly homologous to the PTP1B protein sequence. Homology modeling to reference protein (7lfo.1) was constructed by an online modeling platform to obtain PTP1B protein structure (Swiss-Model, https://swissmodel.expasy.org/, accessed on 28 June 2021). The trisaccharide molecular structure of LPMP was prepared with ChemDraw and then converted into the 3D structure using Chem3D, and the 3D structure was optimized using Avogadro. The PTP1B protein was imported into MOE software, after which the protein was processed (Quick Prepare) and the optimized compound 3D structure was imported into MOE software for molecular docking. Docking studies use the calculated binding energy as an evaluation indicator.

### 4.6. Cell Culture and Insulin Resistance Model Construction

HepG2 cells were cultured in minimal essential medium (MEM) (Procell Life Science &Technology Co., Ltd. Wuhan, China) containing a 10% fetal bovine serum (FBS) and 1% penicillin-streptomycin mixture (Gibco, Grand Island, NY, USA) at 37 °C with 5% CO_2_/95% humidified air. A mixture of palmitic acid and BSA (PA/BSA) was prepared as described previously [43]. Briefly, palmitate (Sigma-Aldrich, St. Louis, MO, USA) was dissolved in distilled water at 70 °C to prepare 100 mM of palmitate for use. and fatty acid (FFA)-free bovine serum albumin (Beijing Solarbio Science & Technology Co., Ltd., Beijing, China) solution was dissolved in distilled water. The above two solutions were then mixed and dissolved at 60 °C and then shaken at 37 °C for 2 h to obtain 5 mM of sodium palmitate complex containing 5% FFA-free BSA. Sodium palmitate has been demonstrated to induce insulin resistance in human HepG2 cells [65,66]. To construct an insulin-resistant cell model for subsequent in vitro evaluation experiments, we cultured HepG2 cells for 24 h. Afterward, the cells were treated with various concentrations of PA/BSA mixture solution for 24 h.

### 4.7. Cell Permeability Assay

HepG2 cells were inoculated in 6-well plates (NEST Biotechnology, Wuxi, China) at a density of 6 × 10^5^ cells/well. Furthermore, cells continued to be incubated at 37 °C for 24 h. Then, the cells were treated with 200 μg·mL^−1^ of FITC-labeled LPMP for various periods. Subsequently, cells were washed three times with PBS buffer to remove excess FITC-LPMP outside the cells, and the cells were collected for analysis by Beckman Coulter flow cytometry (Beckman Coulter, Miami, FL, USA) for fluorescence measurements under Ex488/Em520.

### 4.8. Cell Viability Assessment

HepG2 cells were plated at a concentration of 1 × 10^4^ per well in 96-well plates (NEST Biotechnology, Wuxi, China) and incubated for a further 24 h at 37 °C. Next, the cells were treated with the designated concentration of LPMP for 24 h. After treatment, add 10 μL of CCK-8 solution (Sangon Biotech Co., Ltd., Shanghai, China) to per well and continue to incubate for 1.5 h at 37 °C. Finally, the absorbance at 450 nm was measured with Spark 10 M (Tecan Trading AG, Männedorf, Switzerland).

### 4.9. Glucose Consumption Assay and Insulin Sensitivity Assays

HepG2 cells were plated at a concentration of 2 × 10^4^ per well in 96-well plates and incubated for 24 h at 37 °C. Afterward, cells were treated with PA/BSA complex with or without LPMP for 24 h. Then, HepG2 cells were further cultured for 4 h in phenol red-free MEM medium with or without 200 nm human recombinant insulin (Beijing Solarbio Science & Technology Co., Ltd., Beijing, China). Subsequently, the medium was collected and the glucose residue was determined by the glucose oxidase-peroxidase method according to the Trinder reaction [67,68]. The glucose consumption was obtained by subtracting the remaining glucose content from the total glucose content in the culture medium.

### 4.10. Measurement of Intracellular Reactive Oxygen Species Levels

Changes in fluorescence intensity caused by oxidation of the fluorescent probe DCFH-DA (Beyotime Biotechnology, Shanghai, China) were used to evaluate the level of intracellular ROS production. Briefly, HepG2 cells were seeded in 96-well plates at a 2 × 10^4^/well concentration and incubated for a further 24 h at 37 °C. Afterward, cells were treated with PA/BSA complex with or without LPMP for 24 h, and 100 μg·mL^−1^ N-Acetyl-L-cysteine (NAC) was used as a positive control. Next, cells were incubated with the fluorescent probe DCFH-DA solution for 30 min in the dark. Subsequently, cells were washed with PBS buffer to remove the excess probe, and cell lysis solution (100 μL/well) was added to release intracellular DCF. Finally, fluorescence intensity was measured with Spark 10 M at Ex488/Em525 (Tecan Trading AG, Männedorf, Switzerland).

### 4.11. Determination of Triglycerides (TG), Superoxide Dismutase (SOD), and Malonaldehyde (MDA) Content in HepG2 Cells

HepG2 cells were plated at a concentration of 6 × 10^5^ per well in 6-well plates and subsequently incubated for a further 24 h at 37 °C. After treatment with various concentrations of LPMP for 24 h, and the cells were washed and 150 μL of cell lysis solution containing protease inhibitor was added to each well and lysed on ice for 30 min. The cells were collected, blown well, heated at 70 °C for 10 min, and centrifuged. The supernatant was used for subsequent assays. Determined the intracellular triglyceride content with Triglyceride enzyme assay kit E1025 (Applygen Technologies Inc., Beijing, China), and the detailed procedure for the assay is based on the manufacturer’s protocol. Cellular oxidative stress-related biomarkers SOD and MDA were measured using specialized assay kits S0109 and S0131S (Beyotime Biotechnology, Shanghai, China) based on the manufacturer’s instructions. Protein concentrations were measured with a BCA protein assay kit.

### 4.12. Protein Preparation and Western Blot Assay

The total protein was extracted and immunoblotted as previously described [69]. In brief, cells were washed and lysed with SDS-PAGE loading buffer (100 mM Tris-HCl, 200 mM β-mercaptoethanol, 4% sodium dodecyl sulfate (SDS), 0.2% bromophenol blue, 20% glycerol) for 40 min at 4 °C. The protein samples were separated by SDS-PAGE electrophoresis and transferred to nitrocellulose membranes (Beyotime Biotechnology, Shanghai, China). The blots were blocked with 5% BSA for 1.5 h and subsequently incubated with the primary antibody overnight at 4 °C. Additionally, the following primary antibodies were used to detect specific proteins: pIRS-1 (Y632), activating transcription factor 4 (*ATF4*), pJNK (Thr183/Thr185) (Abcam, Cambridge, MA, USA); insulin receptor substrate 1 (IRS-1), protein kinase B (Akt), pAkt (Ser473), c-Jun N-terminal kinase (JNK), NF-κB p65, β-Actin (Cell Signaling Technology, Beverly, MA, USA); and GADD 34/*CHOP*, GRP 78/Bip (Santa Cruz Biotechnology, Dallas, TX, USA). The membrane was subsequently washed with TBST (Tris-buffer containing Tween 20) and co-incubated with alkaline phosphatase (AP)-labeled secondary antibody (Proteintech Group, Chicago, IL, USA) for 1.5 h at room temperature. Finally, the blots were co-incubated with nitro blue tetrazolium (NBT) and 5-Bromo-4-chloro-3-indolyl-phosphate (BCIP) mixture (Absin, Shanghai, China) for 15 min. Identified the relative grey density of the bands using Image J software (National Institutes of Health).

### 4.13. Total RNA Isolation and Quantitative Real-Time RT-PCR Assay

Total RNA extraction was done using the M5 Universal RNA Mini Kit Tissue/Cell RNA Rapid Extraction Kit (Mei5 Biotechnology Co., Ltd., Beijing, China). The concentration of total RNA was determined with NanoDrop (Thermo Fisher Scientific Incorporated, Waltham, MA, USA) based on the manufacturer’s protocol. cDNA synthesis was evaluated using standard kits (TOYOBO Ltd., Osaka, Japan), following the protocols provided by the manufacturer. RT-PCR analysis was conducted on a BioRad IQ™5 thermal cycler (Bio-Rad, Hercules, CA, USA) with ThunderBird^®^ SYBR^®^ qPCR Mix (TOYOBO Ltd., Osaka, Japan). The cycle threshold (Ct) values were normalized to the *β-actin* gene as the loading control. Relative gene expression levels were calculated with 2^−ΔΔCt^. Each experiment was repeated in triplicate. The primer sets (Sangon Biotech Co., Ltd., Shanghai, China) used in this study are listed in Table 2.

### 4.14. Animal Experiments

The experimental protocol was performed following the ethical standards of ARRIVE guidelines, and efforts were made to minimize animal suffering. Animal experiments were approved by the Ethics and Animal Welfare Committee of the Ocean University of China (OUC-SMP-2020-10-1).

Briefly, 18~22 g male Kunming mice were acquired from Jinan Pengyue Laboratory Animal Co., Ltd. (Jinan, China). All animals were housed at 25 ± 1 °C, 50–75% relative humidity, on a regular light/dark cycle of 12 h. Moreover, all mice were allowed free access to regular chow and water. After 2 weeks of adaptation, the 40 mice were randomly divided into a normal feeding group (8 mice on normal chow) and a high fat and high sugar feeding group (32 mice on high fat and high sugar chow). After 4 weeks of feeding, mice were fasted for 12 h and given three repeated injections of 1.5 mL of fresh streptozocin (STZ) solution (50 mg/kg bodyweight for 3 days) in the high sugar and fat feeding group, and 1.5 mL of saline in the normal feeding group. After 1 week of feeding, mice in the high glucose and high-fat feeding group with fasting blood glucose above 11.1 mmol/L and typical diabetic symptoms such as polydipsia and polyuria were considered type 2 diabetic mice. In total, 32 type 2 diabetic mice were randomly and equally divided into four groups as follows: (1) model group, given with saline; (2) Metf group, supplied with 225 mg/kg/day metformin; (3) LPMP high dosage group (H group), administered with 100 mg/kg/day LPMP; (4) LPMP low dosage group (L group), treated with 20 mg/kg/day LPMP. The mice were administered by gavage once daily for 4 weeks, and equal amounts of saline were given to the control and model groups. 

### 4.15. Biochemical Assays

The insulin tolerance test (ITT) was performed as described [70], and blood glucose levels were measured using a standard blood glucose meter (Johnson & Johnson, New Brunswick, NJ, USA). Triglycerides (TG), total cholesterol (TC), glycosylated hemoglobin (HbA1c), and glucose (GLU) were tested with a standard assay kit (Nanjing Jiancheng Institute of Biological Engineering, Nanjing, China). Alanine aminotransferase (AST) and aspartate aminotransferase (ALT) are important biomarkers of liver damage which were conducted with specific kits (Beijing Solarbio Science & Technology Co., Ltd., Beijing, China).

### 4.16. Histopathological Analysis

Mice were executed after 4 weeks of treatment. Fresh liver and adipose tissues were taken, fixed in 4% paraformaldehyde, and paraffin-embedded tissue was cut to 5 μm sections. The tissue sections were then stained with hematoxylin and eosin (H&E) to determine histomorphology changes. Tissue sections were observed using a high magnification microscope.

### 4.17. Statistical Analysis

All data are expressed as mean ± standard error of the mean (SEM). One-way analysis of variance (ANOVA) was used to statistically analyze the differences in data between the groups. *p* < 0.05 was considered a statistically significant difference. Analyses were performed using GraphPad Prism software (San Diego, CA, USA).

## Figures and Tables

**Table 1:** The ^13^C-NMR date of LPM and LPMP.

Sample	NMR Data (ppm)							
	C_1_	C_2_	C_3_	C_4_	C_5_	C_6_	C_3_-P	C_2_-P
LPM	99.91	69.92	71.30	77.76	75.77	175.15	/	/
LPMP	100.05	69.94	71.15	77.63	75.84	175.18 173.00	73.76	74.83

**Table 2:** Primers designed for quantitative amplification.

Genes	Forward Primer (5′-3′)	Reverse Primer (5′-3′)
*β-actin*	CATGTACGTTGCTATCCAGGC	CTCCTTAATGTCACGCACGAT
*CHOP*	GGAAACAGAGTGGTCATTCCC	CTGCTTGAGCCGTTCATTCTC
*ATF4*	TCCCATCTCCAGGTGTTCTC	CAGCTCTTTGCACTCACCAG
*PTPN1*	AGCCAGTGACTTCCCATGTAG	TGTTGAGCATGACGACACCC
*Xbp1s*	CTGAGTCCGCAGCAGGTG	GTCCAGAATGCCCAACAGGA
*Xbp1u*	CGGAAGCCAAGGGGAATGAA	TGTTCTGGAGGGGTGACAAC

## Data Availability

Not applicable.

## Supplementary Materials

The following are available online at https://www.mdpi.com/article/10.3390/ijms222312693/s1.

## References

[1] Cho N., Shaw J.E., Karuranga S., Huang Y.D., da Rocha Fernandes J.D., Ohlrogge A.W., Malanda B. (2018). IDF Diabetes Atlas: Global estimates of diabetes prevalence for 2017 and projections for 2045. Diabetes Res. Clin. Pract..

[2] Kahn S., Hull R., Utzschneider K. (2006). Mechanisms linking obesity to insulin resistance and type 2 diabetes. Nature.

[3] Sun X.J., Miralpeix M., Myers M.G., Glasheen E.M., Backer J.M., Kahn C.R., White M.F. (1992). Expression and function of IRS-1 in insulin signal transmission. J. Biol. Chem..

[4] Honma M., Sawada S., Ueno Y., Murakami K., Yamada T., Gao J., Kodama S., Izumi T., Takahashi K., Tsukita S. (2018). Selective insulin resistance with differential expressions of IRS-1 and IRS-2 in human NAFLD livers. Int. J. Obes..

[5] Molinaro A., Becattini B., Mazzoli A., Bleve A., Radici L., Maxvall I., Sopasakis V.R., Molinaro A., Backhed F., Solinas G. (2019). Insulin-Driven PI3K-AKT Signaling in the Hepatocyte Is Mediated by Redundant PI3Kalpha and PI3Kbeta Activities and Is Promoted by RAS. Cell Metab..

[6] Johnson T.O., Ermolieff J., Jirousek M.R. (2002). Protein tyrosine phosphatase 1B inhibitors for diabetes. Nat. Rev. Drug Discov..

[7] Yaribeygi H., Sathyapalan T., Atkin S.L., Sahebkar A. (2020). Molecular Mechanisms Linking Oxidative Stress and Diabetes Mellitus. Oxid. Med. Cell. Longev..

[8] Cang X., Wang X., Liu P., Wu X., Yan J., Chen J., Wu G., Jin Y., Xu F., Su J. (2016). PINK1 alleviates palmitate induced insulin resistance in HepG2 cells by suppressing ROS mediated MAPK pathways. Biochem. Biophys. Res. Commun..

[9] Găman M.A., Epîngeac M.E., Diaconu C.C., Găman A.M. (2020). Evaluation of oxidative stress levels in obesity and diabetes by the free oxygen radical test and free oxygen radical defence assays and correlations with anthropometric and laboratory parameters. World J. Diabetes.

[10] Sarvani C., Sireesh D., Ramkumar K.M. (2017). Unraveling the role of ER stress inhibitors in the context of metabolic diseases. Pharm. Res..

[11] Ajoolabady A., Wang S., Kroemer G., Klionsky D.J., Uversky V.N., Sowers J.R., Aslkhodapasandhokmabad H., Bi Y., Ge J., Ren J. (2021). ER stress in Cardiometabolic Diseases: From Molecular Mechanisms to Therapeutics. Endocr. Rev..

[12] Özcan U., Cao Q., Yilmaz E., Lee A.H., Iwakoshi N.N., Özdelen E., Tuncman G., Görgün C., Glimcher L.H., Hotamisligil G.S. (2004). Endoplasmic Reticulum Stress Links Obesity, Insulin Action, and Type 2 Diabetes. Science.

[13] Dube N., Tremblay M.L. (2005). Involvement of the small protein tyrosine phosphatases TC-PTP and PTP1B in signal transduction and diseases: From diabetes, obesity to cell cycle, and cancer. Biochim. Biophys. Acta.

[14] Stuible M., Tremblay M.L. (2010). In control at the ER: PTP1B and the down-regulation of RTKs by dephosphorylation and endocytosis. Trends Cell Biol..

[15] Yudushkin I.A., Schleifenbaum A., Kinkhabwala A., Neel B.G., Schultz C., Bastiaens P.I. (2007). Live-Cell Imaging of Enzyme-Substrate Interaction Reveals Spatial Regulation of PTP1B. Science.

[16] Haj F., Verveer P., Squire A., Neel B., Bastiaens P. (2002). Imaging Sites of Receptor Dephosphorylation by PTP1B on the Surface of the Endoplasmic Reticulum. Science.

[17] Salmeen A., Andersen J.N., Myers M.P., Tonks N.K., Barford D. (2000). Molecular Basis for the Dephosphorylation of the Activation Segment of the Insulin Receptor by Protein Tyrosine Phosphatase 1B. Mol. Cell.

[18] Elchebly M., Payette P., Michaliszyn E., Cromlish W., Collins S., Loy A.L., Normandin D., Cheng A., Himms-Hagen J., Chan C.C. (1999). Increased insulin sensitivity and obesity resistance in mice lacking the protein tyrosine phosphatase-1B gene. Science.

[19] Van Huijsduijnen R.H., Bombrun A., Swinnen D. (2002). Selecting protein tyrosine phosphatases as drug targets. Drug Discov. Today.

[20] Park H., Bhattarai B.R., Ham S.W., Cho H. (2009). Structure-based virtual screening approach to identify novel classes of PTP1B inhibitors. Eur. J. Med. Chem..

[21] Combs A.P. (2010). Recent advances in the discovery of competitive protein tyrosine phosphatase 1B inhibitors for the treatment of diabetes, obesity, and cancer. J. Med. Chem..

[22] Panikkar R., Brasch D.J. (1996). Composition and block structure of alginates from New Zealand brown seaweeds. Carbohydr. Res..

[23] Haug A., Larsen B., Smidsrod O., Møller J., Brunvoll J., Bunnenberg E., Djerassi C., Records R. (1996). A Study of the Constitution of Alginic Acid by Partial Acid Hydrolysis. Acta Chem. Scand..

[24] Heyraud A., Gey C., Leonard C., Rochas C., Girond S., Kloareg B. (1996). NMR spectroscopy analysis of oligoguluronates and oligomannuronates prepared by acid or enzymatic hydrolysis of homopolymeric blocks of alginic acid. Application to the determination of the substrate specificity of Haliotis tuberculata alginate lyase. Carbohydr. Res..

[25] Fan Y., Li Y., Zhang J., Ding X., Cui J., Wang G., Wang Z., Wang L. (2019). Alginate Enhances Memory Properties of Antitumor CD8^+^ T Cells by Promoting Cellular Antioxidation. ACS Biomater. Sci. Eng..

[26] Son E.W., Rhee D.K., Pyo S. (2003). Antiviral and tumoricidal activities of alginate-stimulated macrophages are mediated by different mechanisms. Arch. Pharm. Res..

[27] Meng X., Li T., Song T., Chen C., Venkitasamy C., Pan Z., Zhang H. (2019). Solubility, structural properties, and immunomodulatory activities of rice dreg protein modified with sodium alginate under microwave heating. Food Sci. Nutr..

[28] Xing M., Cao Q., Wang Y., Xiao H., Zhao J., Zhang Q., Ji A., Song S. (2020). Advances in Research on the Bioactivity of Alginate Oligosaccharides. Mar. Drugs.

[29] Yang J.H., Bang M.A., Jang C.H., Jo G.H., Jung S.K., Ki S.H. (2015). Alginate oligosaccharide enhances LDL uptake via regulation of LDLR and PCSK9 expression. J. Nutr. Biochem..

[30] Zheng J., Li H., Zhang X., Jiang M., Luo C., Lu Z., Xu Z., Shi J. (2018). Prebiotic Mannan-Oligosaccharides Augment the Hypoglycemic Effects of Metformin in Correlation with Modulating Gut Microbiota. J. Agric. Food Chem..

[31] Wang H., Zhang X., Wang S., Li H., Lu Z., Shi J., Xu Z. (2018). Mannan-oligosaccharide modulates the obesity and gut microbiota in high-fat diet-fed mice. Food Funct..

[32] Wang Y., Li L., Ye C., Yuan J., Qin S. (2020). Alginate oligosaccharide improves lipid metabolism and inflammation by modulating gut microbiota in high-fat diet fed mice. Appl. Microbiol. Biotechnol..

[33] Bi D., Xiao S., Lin Z., Yao L., Fang W., Wu Y., Xu H., Lu J., Xu X. (2021). Alginate-Derived Mannuronate Oligosaccharide Attenuates Tauopathy through Enhancing Autophagy. J. Agric. Food Chem..

[34] Shafiq S. (2019). Genetic, structural and pharmacological characterization of polymannuronate synthesized by algG mutant indigenous soil bacterium *Pseudomonas aeruginosa* CMG1421. J. Appl. Microbiol..

[35] Li Q., Zeng Y., Wang L., Guan H., Li C., Zhang L. (2017). The heparin-like activities of negatively charged derivatives of low-molecular-weight polymannuronate and polyguluronate. Carbohydr. Polym..

[36] Coleman R.J., Lawrie G., Lambert L.K., Whittaker M., Jack K.S., Grondahl L. (2011). Phosphorylation of alginate: Synthesis, characterization, and evaluation of in vitro mineralization capacity. Biomacromolecules.

[37] Liu G.X., Tan J.Z., Niu C.Y., Shen J.H., Luo X.M., Shen X., Chen K.X., Jiang H.L. (2006). Molecular dynamics simulations of interaction between protein-tyrosine phosphatase 1B and a bidentate inhibitor. Acta Pharm. Sin..

[38] Chen X., Liu X., Gan Q., Feng C., Zhang Q. (2019). Molecular Dynamics Simulations of A27S and K120A Mutated PTP1B Reveals Selective Binding of the Bidentate Inhibitor. Biomed. Res. Int..

[39] Peters G.H., Iversen L.F., Andersen H.S., Møller N.P.H., Olsen O.H. (2004). Residue 259 in protein-tyrosine phosphatase PTP1B and PTPalpha determines the flexibility of glutamine 262. Biochemistry.

[40] Stuible M., Doody K.M., Tremblay M.L. (2008). PTP1B and TC-PTP: Regulators of transformation and tumorigenesis. Cancer Metastasis Rev..

[41] Li X., Wang L., Shi D. (2016). The design strategy of selective PTP1B inhibitors over TCPTP. Bioorg. Med. Chem..

[42] Xu Q., Luo J., Wu N., Zhang R., Shi D. (2018). BPN, a marine-derived PTP1B inhibitor, activates insulin signaling and improves insulin resistance in C2C12 myotubes. Int. J. Biol. Macromol..

[43] Wang X., Jiang H., Zhang N., Cai C., Li G., Hao J., Yu G. (2020). Anti-diabetic activities of agaropectin-derived oligosaccharides from Gloiopeltis furcata via regulation of mitochondrial function. Carbohydr. Polym..

[44] Arner P., Rydén M. (2015). Fatty Acids, Obesity and Insulin Resistance. Obes. Facts.

[45] Boden G., Shulman G.I. (2002). Free fatty acids in obesity and type 2 diabetes: Defining their role in the development of insulin resistance and beta-cell dysfunction. Eur. J. Clin. Investig..

[46] Bakhtiyari S., Meshkani R., Taghikhani M., Larijani B., Adeli K. (2010). Protein tyrosine phosphatase-1B (PTP-1B) knockdown improves palmitate-induced insulin resistance in C2C12 skeletal muscle cells. Lipids.

[47] Khamzina L., Gruppuso P.A., Wands J.R. (2003). Insulin signaling through insulin receptor substrate 1 and 2 in normal liver development. Gastroenterology.

[48] Matsuda M., Shimomura I. (2013). Increased oxidative stress in obesity: Implications for metabolic syndrome, diabetes, hypertension, dyslipidemia, atherosclerosis, and cancer. Obes. Res. Clin. Pract..

[49] Wang Y., Branicky R., Noe A., Hekimi S. (2018). Superoxide dismutases: Dual roles in controlling ROS damage and regulating ROS signaling. J. Cell Biol..

[50] Bacanlı M., Anlar H.G., Aydın S., Çal T., Arı N., Bucurgat Ü.Ü., Başaran A.A., Başaran N. (2017). D-limonene ameliorates diabetes and its complications in streptozotocin-induced diabetic rats. Food Chem. Toxicol..

[51] Hirosumi J., Tuncman G., Chang L., Görgün C.Z., Uysal K.T., Maeda K., Hotamisligil G.S. (2002). A central role for JNK in obesity and insulin resistance. Nature.

[52] Ha S.Y., Qiu X.M., Lai Z.Z., Yang H.L., Wang Y., Ruan L.Y., Shi J.W., Zhu X.Y., Li D.J., Li M.Q. (2020). Excess palmitate induces decidual stromal cell apoptosis via the TLR4/JNK/NF-kB pathways and possibly through glutamine oxidation. Mol. Hum. Reprod..

[53] Sadeghi A., Rostamirad A., Seyyedebrahimi S., Meshkani R. (2018). Curcumin ameliorates palmitate-induced inflammation in skeletal muscle cells by regulating JNK/NF-kB pathway and ROS production. Inflammopharmacology.

[54] Nakatani Y., Kaneto H., Kawamori D., Hatazaki M., Miyatsuka T., Matsuoka T.A., Kajimoto Y., Matsuhisa M., Yamasaki Y., Hori M. (2004). Modulation of the JNK pathway in liver affects insulin resistance status. J. Biol. Chem..

[55] Jiang S., Messina J.L. (2011). Role of inhibitory kappaB kinase and c-Jun NH_2_-terminal kinase in the development of hepatic insulin resistance in critical illness diabetes. Am. J. Physiol.-Gastrointest. Liver Physiol..

[56] Cai D., Yuan M., Frantz D.F., Melendez P.A., Hansen L., Lee J., Shoelson S.E. (2005). Local and systemic insulin resistance resulting from hepatic activation of IKK-beta and NF-kappaB. Nat. Med..

[57] Barma P., Bhattacharya S., Bhattacharya A., Kundu R., Dasgupta S., Biswas A., Bhattacharya S., Roy S.S., Bhattacharya S. (2009). Lipid induced overexpression of NF-kappaB in skeletal muscle cells is linked to insulin resistance. Biochim. Biophys. Acta.

[58] Cao J., Dai D.L., Yao L., Yu H.H., Ning B., Zhang Q., Chen J., Cheng W.H., Shen W., Yang Z.X. (2012). Saturated fatty acid induction of endoplasmic reticulum stress and apoptosis in human liver cells via the *PERK*/*ATF4*/*CHOP* signaling pathway. Mol. Cell Biochem..

[59] Kim D.S., Jeong S.K., Kim H.R., Kim D.S., Chae S.W., Chae H.J. (2010). Metformin regulates palmitate-induced apoptosis and ER stress response in HepG2 liver cells. Immunopharmacol. Immunotoxicol..

[60] Wang Y., Liang K., Kong W. (2019). Intestinal Trefoil Factor 3 Alleviates the Intestinal Barrier Function Through Reducing the Expression of TLR4 in Rats with Nonalcoholic Steatohepatitis. Arch. Med. Res..

[61] Egbuna C., Awuchi C.G., Kushwaha G., Rudrapal M., Patrick-Iwuanyanwu K.C., Singh O., Odoh U.E., Khan J., Jeevanandam J., Kumarasamy S. (2021). Bioactive Compounds Effective Against Type 2 Diabetes Mellitus: A Systematic Review. Curr. Top. Med. Chem..

[62] Li Q., Li C., Yang C., Liu C., Yu G., Guan H. (2013). Preparation, characterization and antioxidant activities of polymannuronic acid phosphate, H-phosphonate and sulfate. Int. J. Biol. Macromol..

[63] Yang Y., Zhao X., Li J., Jiang H., Shan X., Wang Y., Ma W., Hao J., Yu G. (2018). A beta-glucan from Durvillaea Antarctica has immunomodulatory effects on RAW264.7 macrophages via toll-like receptor 4. Carbohydr. Polym..

[64] Harris W.D., Popat P. (1954). Determination of the phosphorus content of lipids. J. Am. Oil Chem. Soc..

[65] Lee J.Y., Cho H.K., Kwon Y.H. (2010). Palmitate induces insulin resistance without significant intracellular triglyceride accumulation in HepG2 cells. Metabolism.

[66] Ishii M., Maeda A., Tani S., Akagawa M. (2015). Palmitate induces insulin resistance in human HepG2 hepatocytes by enhancing ubiquitination and proteasomal degradation of key insulin signaling molecules. Arch. Biochem. Biophys..

[67] Trinder P. (1969). Determination of blood glucose using an oxidase-peroxidase system with a non-carcinogenic chromogen. J. Clin. Pathol..

[68] Barham D., Trinder P. (1972). An improved colour reagent for the determination of blood glucose by the oxidase system. Analyst.

[69] Lu X., Geng J., Zhang J., Miao J., Liu M. (2019). Xanthohumol, a Prenylated Flavonoid from Hops, Induces Caspase-Dependent Degradation of Oncoprotein BCR-ABL in K562 Cells. Antioxidants.

[70] Du J., Zhao Y.T., Wang H., Zhang L.X., Qin G., Zhuang S., Kadin M., Chin Y.E., Liu P.Y., Zhao T.C. (2021). The Essential Role of PRAK in Preserving Cardiac Function and Insulin Resistance in High-Fat Diet-Induced Diabetes. Int. J. Mol. Sci..

