# Supplementary material for: A Novel PTP1B Inhibitor-Phosphate of Polymannuronic Acid Ameliorates Insulin Resistance by Regulating IRS-1/Akt Signaling
Source: Int J Mol Sci. 2021 Nov 24;22(23):12693. doi: 10.3390/ijms222312693 (PMC8657924; doi:10.3390/ijms222312693)
Supplement: Supplementary file 1 [file ijms-22-12693-s001.zip › ijms-1465758-supplementary.pdf]

supplementary

A Novel PTP1B Inhibitor-phosphate of Polymannuronic Acid Ameliorates Insulin Resistance by Regulating IRS-1/Akt Signaling

Dan Li [1, #], Shuai Zhang [1, #], Cheng Yang [1], Quancai Li [1, 3], Shixin Wang [1, 3], Ximing Xu [1, 2, 3], Jiejie Hao [1, 2] * and Chunxia Li [1,2,3,]*

1. Key Laboratory of Marine Drugs of Ministry of Education, Shandong Provincial Key Laboratory of Glycoscience and Glycoengineering, School of Medicine and Pharmacy, Ocean University of China, Qingdao 266003, China; ldan@stu.ouc.edu.cn (D.L.); 21170831086@stu.ouc.edu.cn (S.Z.); acheng0912@163.com (C.Y.); quancaili@126.com (Q.L.); shixin113@126.com (S.W.); xuximing@ouc.edu.cn (X.X.)
2. Laboratory for Marine Drugs and Bioproducts of Pilot National Laboratory for Marine Science and Technology (Qingdao), Qingdao 266237, China
3. Laboratory of Marine Glycodrug Research and Development, Marine Biomedical Research Institute of Qingdao, Qingdao 266071, China

\# These authors have contributed equally to this work

* Correspondence: 2009haojie@ouc.edu.cn (J.H.); lchunxia@ouc.edu.cn (C.L.). Tel: +86-532-8203-2030. Fax: +86-532-8203-3054.

Supplementary Method:

Determination of FITC labeling rate

The FITC standard stock solution was prepared at a 250 μg/mL concentration, and the standard stock solution was diluted to a series of standard solutions at concentrations of 0, 0.250, 0.500, 0.625 1.250, and 2.500 μg/mL, respectively. The fluorescence intensities of the FITC standard solution were measured at λ_{ex} = 494 nm and λ_{em} = 545 nm using PBS buffer solution (0.01 mol/L) as the blank control. The concentration-fluorescence intensity standard curve was plotted. An appropriate amount of LPMP-FITC was weighed and dissolved in PBS buffer solution (0.01 mol/L) to prepare a sample solution with a concentration of 150 μg/ml LPMP-FITC. The labeling rate of LPMP-FITC was calculated according to the standard curve.

Result:

Result for FITC labeling rate

The concentration-fluorescence intensity standard curve was shown in Fig. S1., and the calculated FITC labeling rate of LPMP-FITC was 2.3‰.

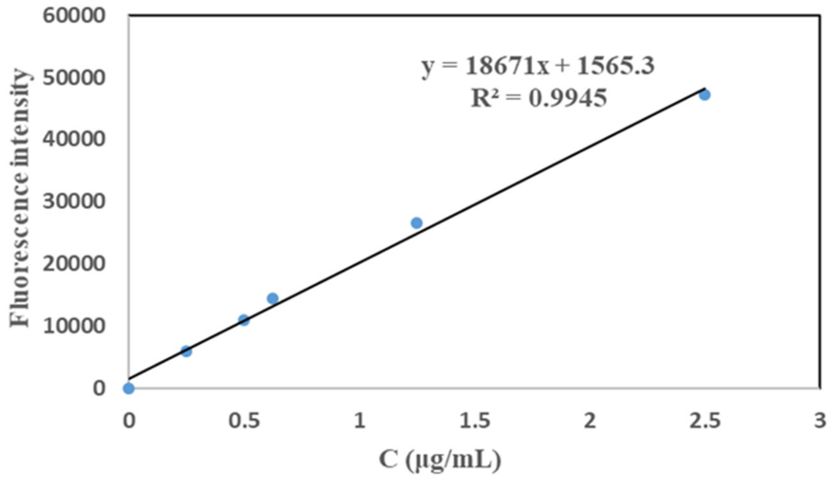

Figure S1. The concentration-fluorescence intensity standard curve.
